# Sexual and Natural Selection Both Influence Male Genital Evolution

**DOI:** 10.1371/journal.pone.0063807

**Published:** 2013-05-22

**Authors:** Clarissa M. House, Zenobia Lewis, Dave J. Hodgson, Nina Wedell, Manmohan D. Sharma, John Hunt, David J. Hosken

**Affiliations:** Centre for Ecology and Conservation, Biosciences, University of Exeter, Penryn, Cornwall, United Kingdom; Michigan State University, United States of America

## Abstract

Rapid and divergent evolution of male genital morphology is a conspicuous and general pattern across internally fertilizing animals. Rapid genital evolution is thought to be the result of sexual selection, and the role of natural selection in genital evolution remains controversial. However, natural and sexual selection are believed to act antagonistically on male genital form. We conducted an experimental evolution study to investigate the combined effects of natural and sexual selection on the genital-arch lobes of male *Drosophila simulans*. Replicate populations were forced to evolve under lifetime monogamy (relaxed sexual selection) or lifetime polyandry (elevated sexual selection) and two temperature regimes, 25°C (relaxed natural selection) or 27°C (elevated natural selection) in a fully factorial design. We found that natural and sexual selection plus their interaction caused genital evolution. Natural selection caused some aspects of genital form to evolve away from their sexually selected shape, whereas natural and sexual selection operated in the same direction for other shape components. Additionally, sexual and natural selection tended to favour larger genitals. Thus we find that the underlying selection driving genital evolution is complex, does not only involve sexual selection, and that natural selection and sexual selection do not always act antagonistically.

## Introduction

The extreme diversity of male genital morphology across animals with internal fertilization is a conspicuous and general trend [1]. Historically natural selection was invoked to explain male genital evolution, but the current consensus is that sexual selection is primarily responsible for this rapid, divergent evolution [1]–[3]. Compelling evidence for this comes from comparative work showing that genitals are more complex and evolve more rapidly in species with elevated post-copulatory sexual selection [3]. Experimental evolution studies have documented similar patterns at a micro-evolutionary scale, with sexual selection generating size and shape changes in the genitalia of male and female dung beetles, *Onthophagus taurus* [4], [5], as well as evolution of the static allometry of male genital spines and two non-intromittent genital traits in the seed beetle, *Callosobruchus maculatus* [6]. Nonetheless, there is a paucity of direct experimental studies that unequivocally demonstrate genital evolution through sexual selection.

Despite the evidence that genitals evolve through sexual selection, the phenotypic and genetic variation in genital traits is unusually low [7], [8], [9], [10], [11], [12], which has been attributed to strong stabilizing selection on genital morphology to prevent interspecies mating [13] or for male genital morphology to ‘fit’ the average conspecific female [8], [14]. In addition, the primary function of male genitalia is to transfer sperm to females, so the genitals must be of an appropriate size and shape to facilitate ejaculate conveyance. Thus, natural selection may also play an important role in male genital evolution [15], which could result in antagonistic natural and sexual selection acting on genital morphology [16], thereby generating net stabilizing selection. However, the combined influences of natural and sexual selection acting on genitalia have rarely been investigated empirically [6], [17], [18] and claims that natural selection acts on genital form remain extremely controversial [1], [2], [14], [19].

One explanation for the lack of empirical data is that natural and sexual selection frequently co-occur so that it can be difficult to disentangle which mode of selection is responsible for the phenotypes that are observed [20]. Furthermore, both natural and sexual selection generate similar fitness outcomes, that is, some individuals in the population will have greater fitness than others. Despite the inherit difficulty in distinguishing between the relative importance of these two modes of selection, experimental evolution studies are invaluable for determining whether natural and sexual selection and/or their interaction are antagonistic or favour the same ‘optimal’ phenotype [21].

Here we use experimental evolution to investigate the combined effects of natural and sexual selection on the size and shape of the posterior and ventral lobes of the *Drosophila simulans* genital arch. The genital arch of *Drosophila* is strikingly variable and is a key diagnostic structure used in species identification. For example, *D. simulans* is easily differentiated from *D. melanogaster* using this trait, even though the two species are otherwise extremely difficult to distinguish [22], [23]. Time-sequence functional analyses suggest that the *Drosophila* genital arch aids in grasping the female genitalia and establishing genital coupling during copulation [24]. The importance of the genital arch for successful coupling and insemination is also implied from QTL analysis, which suggests a history of consistent directional selection on the trait [25]. We compared the morphology of the posterior and ventral lobes of the male genital arch of flies from replicate populations that had been evolving for 47 generations under lifetime monogamy (each male paired with one female = relaxed sexual selection) or lifetime polyandry (each female paired with four males = elevated sexual selection). To determine whether the responses to relaxed and elevated sexual selection were influenced by natural selection we also imposed two temperature regimes, 25°C or 27°C in a fully factorial design. 25°C, the ancestral temperature to which flies had adapted for more than 140 generations in our laboratory will reflect relaxed natural selection, especially since 27°C, the high temperature treatment is close to the sterility level of the flies and increases the expression of dessication proofing CHCs [26], which is clearly indicative of relatively elevated natural selection. This generated four experimental treatments: (i) relaxed natural and sexual selection (-N-S), (ii) relaxed natural and elevated sexual selection (-N+S), (iii) elevated natural and relaxed sexual selection (+N-S) and (iv) elevated natural and sexual selection (+N+S). We found that both forms of selection influenced the evolution of genital size and shape and sometimes, but not always, seemed to act antagonistically.

## Materials and Methods

### Experimental Animals

The laboratory populations of *D. simulans* were derived from twenty iso-female lines supplied by the Centre for Environmental Stress and Adaptation Research, La Trobe University, Australia, that had been mixed in population cages. Populations have been maintained in cages at 25°C under a 12∶12 h light:dark cycle with ca. 800–1000 flies per cage with overlapping generations and free mate choice for more than 4 years prior to the establishment of experimental populations and harbour substantial genetic variation for every trait examined so far [27], [28]. Flies were reared under the same conditions in standard vials on ‘Drosophila quick mix medium’ (Blades Biological, Edenbridge, Kent, U.K.), nipagin and water.

### Selection Regime

Experimental evolution populations (*n* = 16) were established to explore the response of male genital morphology to elevated (+) and relaxed (-) sexual (S) and natural (N) selection. The design was fully factorial, with 4 treatment combinations (-N-S, -N+S, +N-S, +N+S) with 4 replicate populations per treatment [26]. Flies had been reared at 25°C for ca. 140 generations, so this represents the relaxed natural selection (-N) treatment and rearing temperature of 27°C represented the elevated natural selection (+N) treatment. At 27°C males are close at their sterility threshold (i.e. this is close to a population extinction threshold) and flies are of elevated risk of desiccation [26] so this is a stressful temperature which results in elevated natural selection relative to 25°C. Female *D. simulans* control mating and mate infrequently across their life span (typically twice), irrespective of whether mating is with the same or different males [29], [30]. With respect to our experimental design, females were housed singularly with one male (monogamy) to remove the opportunity for female mate choice and therefore relax sexual selection (-S) or females were housed singularly with four males (polyandry) to increase the opportunity for pre- and post-copulatory mate choice and therefore elevate sexual selection (+S). To approximately standardise effective population size and eliminate potential differential inbreeding, we had 60 females per population in the elevated sexual selection treatment and 64 females per population in the relaxed sexual selection treatment [26]. As females re-mate relatively infrequently and sperm displacement is ca. 80% we calculated that 4 additional pairs were sufficient to standardize the effective population size (Ne) [26]. This is true even if female mating rate evolves. Following 47 generations of experimental evolution under the different treatments, male genital morphology was assayed.

**Figure 1:**
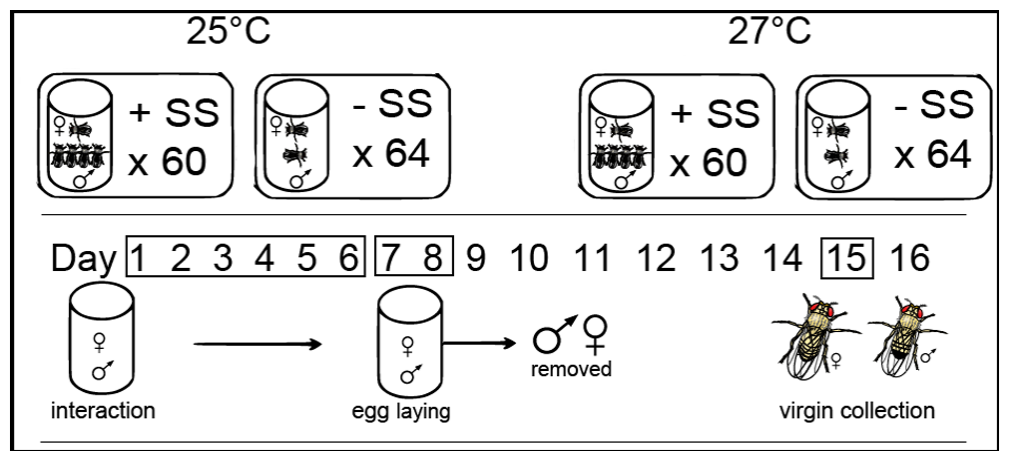
A diagrammatic representation of the protocol used in the experimental evolution. Females and males were housed for 6 days (1–6) in interaction vials before they were moved to egg-laying vials for two days (days 7 & 8). Adults were then discarded. Eggs in egg-laying vials were allowed to develop and individuals emerging from these started subsequent generations (virgin collection on day 15).

In brief, the protocol for the maintenance of the selection lines is as follows. The replicate populations for each selection line were split between two incubators (i.e. two populations/per selection line/per incubator so that there were a total of eight populations in each of two incubators set to the different temperatures). Flies were housed for 6 days in ‘survival vials’ and then transferred to ‘egg laying vials’ for 2 days. Food was provided in excess (>40 ml/vial maximises offspring emergence rates) to minimise differential development and mortality due to larval competition. The adults were discarded and the vials were incubated until peak offspring emergence (ca. 9 days after egg laying). To ensure virginity, flies that eclosed overnight were killed and virgins were collected ca. 7 hrs after. Virgin offspring from our replicate populations were pooled by sex within each selection line. Within these groupings, individuals were randomly selected to commence to the next generation (figure 1, [26]).

After 47 generations, mixed sex pairs (n = 20) from each line were allowed to mate and oviposit for 24 hr in ‘egg laying’ vials at 26°C to standardize any potential maternal effects across our selection lines. The development of individuals from all lines was standardised by incubating the vials at 26°C also. Thus any divergence in the lines could be attributed to evolutionary not developmental conditions. Six days after the eclosion of the first offspring per vial, offspring were collected and frozen. This ensured that all offspring had eclosed and the cuticular structures of the genitalia had hardened. A random subset of 10 male offspring from each population was sampled and the population mean of each morphological measure was calculated so that all analyses employed line means.

### Morphometrics

Male genitalia were separated from the abdomen and soaked in a drop of 50∶50 glycerol and lactic acid for 60 minutes, which softens and clears the tissues. The genital arch is a delicate, paired structure that is prone to damage during dissection. Therefore, the intact genital arch (be it the left or right) was oriented consistently and mounted using Hoyer’s solution. In addition, one non-sexual trait, the left or right hind tibia was randomly selected and removed from the thorax and mounted using Hoyer’s solution also. Digital images of the genitalia and hind tibia were captured using a Leica M125 microscope with mounted camera that conveyed images to a PC. Two measures of the length of the hind tibia, (which was used as an index of body size) was measured using Image J. The repeatability of the measurement is high (r-value = 0.98; *β* = 1.01, n = 25).

**Figure 2:**
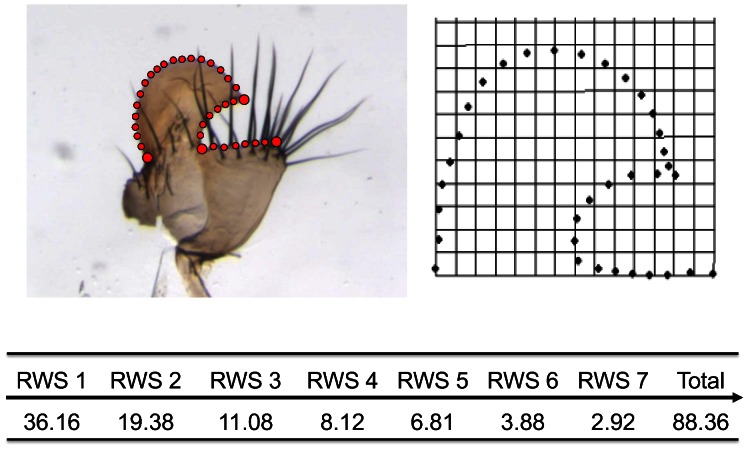
The posterior (P) and ventral (V) lobes of the *D. simulans* gential arch (left) and the consensus genital configuration (right) defined using geometric morphometrics, which quantified the variation in arch size and shape. Large red dots on the left (arch) plate represent Type 2 landmarks (n = 4), smaller red dots (n = 30) are sliding semi-landmarks. The 34 landmarks and semi-landmarks yield 64 Relative Warp Scores (RWS), of which 88.36% of the variance is explained by RWS1–7.

Geometric morphometric analysis was used to quantify the variation in the size and shape of the outline of the posterior and ventral lobe of the genital arch. Four points along the outline that could be located precisely across all specimens were applied as landmarks (type-two landmarks). Another 30 points, called sliding semilandmarks, were allowed to slide along the outline in a trajectory that minimizes shape changes between specimens and the Procrustes average of all the specimens [31] (figure 2). The points (landmarks and semilandmarks) were digitized in TPSDIG 2.14 [32] and the semilandmarks were identified by use of a ‘sliders file’ in TPSUTIL 1.46 [31]. To eliminate non-shape variation, the digitized landmark data were normalized for position, orientation and scale (generalized least squares superimposition). Centroid size, the square root of landmarks from the centroid, was extracted and the data were reduced to a series of relative warp scores. Our 34 landmarks and semilandmarks yield 64 relative warp scores that explain progressively less variance. Beyond RWS 7, less than 2% of the variance in shape was distinctly explained, so we only interpret RWS 1–7 [33] (figure 2). Changes in the shape of the posterior and ventral lobe of the genital arch were visualized as shape deformations of the thin plate spline. tpsRELW 1.46 was used for the superimposition, calculation of centroid and relative warp scores and thin-plate spline plot visualizations [31]. We assessed the repeatability of digitization by digitizing two images of the same genitals twice (n = 25). Ordinary least squares regression (i.e. RW1 on RW1; RW2 on RW2 etc) revealed that we were able to digitize the genital arches consistently (r-values ranged from 0.95 to 0.64; *β* ranged from 1.06 to 0.79 in RW1–RW7) although, error in RW 1 and subsequent RWs accumulated so that the r-values were lower in RW7 (r-value = 0.64; *β = *0.79).

### Statistical Analyses

ANOVA and multivariate analysis of variance (MANOVA with type III sums of squares) were performed, with the natural selection treatment and sexual selection treatment and their interaction included as fixed effects, to analyse the effects of sexual selection and natural selection on male genital size and shape. Genitals can scale with body size [8] and Pearson’s correlation tests showed that our measure of body size was correlated with centroid size (r-value = 0.561, *P* = 0.024), but the RWS were un-correlated with body size (r-values ranged from = −0.096– −0.385, *P* = 0.723–0.141). To remove any variance in genital size that may have been due to body size we included hind tibia length as a covariate in the genital size analysis. Note, our index of body size (i.e. tibia length) did not differ across our treatments; Sexual selection (S); F_1,15_ = 1.757, *P* = 0.210; Natural selection (N); F_1,15_ = 1.337, *P* = 0.270; S×N; F_1,15_ = 1.210, *P* = 0.293. Centroid size may be correlated with genital shape variables, as shape often changes with size due to allometry [33], and Pearson’s correlation tests indicated that some RWS (RW4, r-value = −0.505, *P* = 0.046; RW7, r-value = −0.615, *P* = 0.011) were significantly correlated with centroid size. To remove any variance in shape that was associated with genital size, we therefore included centroid size as a covariate in shape analyses as recommended [33]. Analyses were performed using SPSS (PASWStatistics 19).

## Results

**Figure 3:**
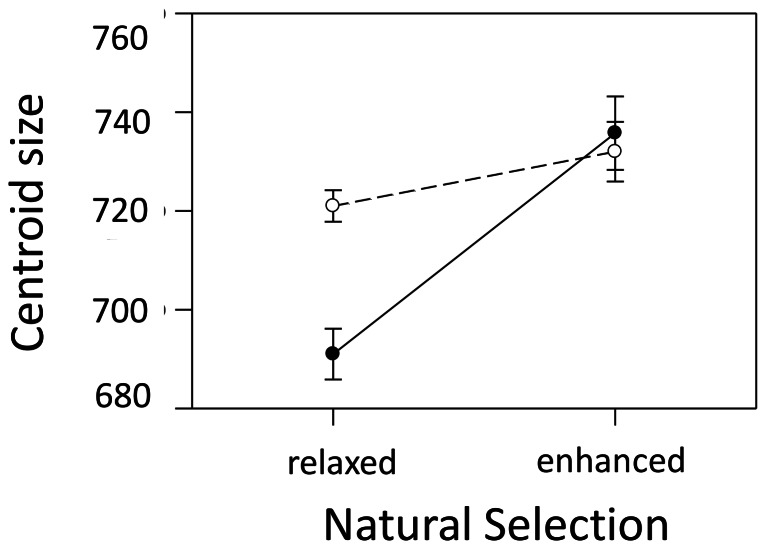
The effect of natural and sexual selection on genital size (measured as centroid size). Mean Centroid Scores for each population (i.e. 4 replicate populations/treatment ) were treated as a line means. Open circles (o) and dashed line equals elevated sexual selection, filled circles (•) and solid line represents relaxed sexual selection (experimentally enforced monogamy). Natural selection (*F*_1,12_ = 19.10; *P* = 0.001) and the interaction between natural and sexual selection (*F*_1,12_ = 6.388; *P = *0.028) significantly influenced the size of the genital arch such that elevated natural selection and the interaction between natural and sexual selection favoured larger genital size.

Natural selection, sexual selection and their interaction all significantly influence the size of male genitals (figure 3). When natural and sexual selection were relaxed (-N-S), genital size was smaller, but in populations with elevated sexual selection (+S), larger genitals were found (figure 3). Elevated natural selection with relaxed sexual selection (+N-S) also favoured large genital size (figure 3), furthermore, the interaction between elevated natural and sexual selection clearly favour significantly larger genitals also.

**Figure 4:**
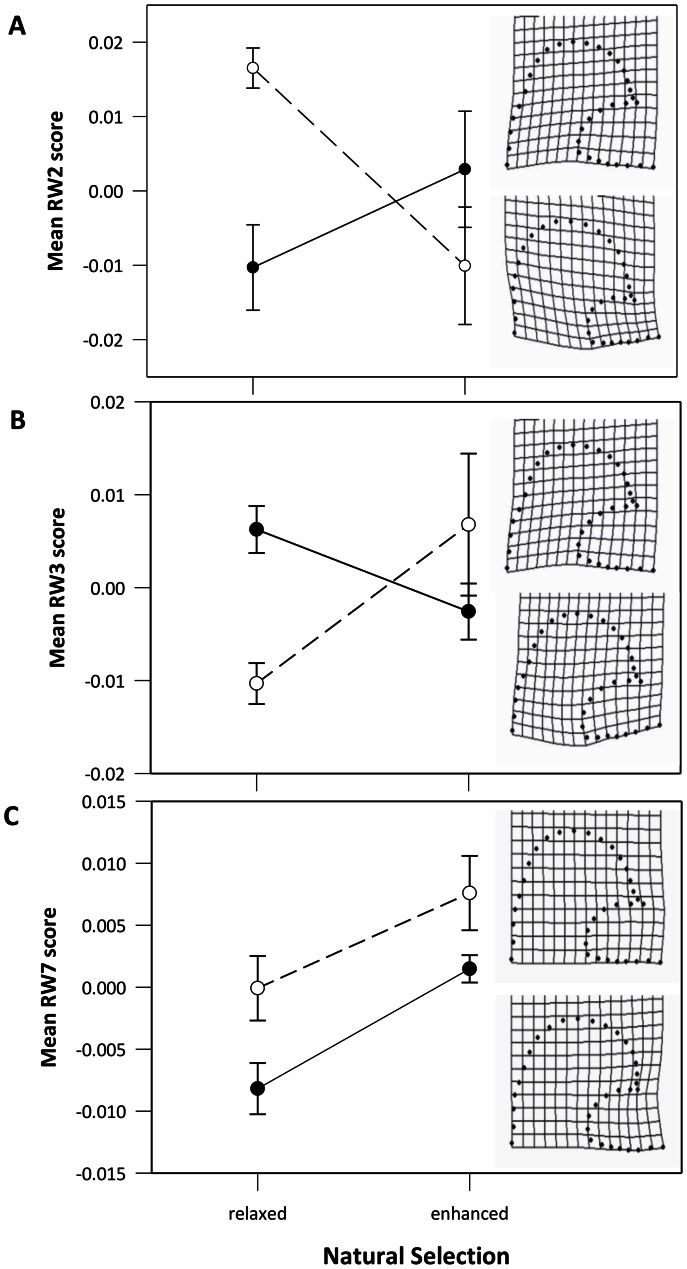
The effect of natural and sexual selection on genital shape, as defined by the Relative Warp (RW) scores. Mean Relative Warp Scores for each population (i.e. 4 replicate populations/treatment ) were treated as a line means. Shown here are the three RWs that were significantly influenced by selection (Table 1). Open circles (o) and dashed line represent the elevated sexual selection treatment, filled circles (•) and solid line represents relaxed sexual selection (experimentally enforced monogamy). Inset figures show the shapes represented by high warp scores on the top inset panel and low warp scores on the bottom inset panel for each Warp. For RW2 and RW3 the interaction between sexual and natural selection significantly influenced shape, while for RW7, the interaction was not significant, but both modes of selection significantly influenced shape (Table 1).

Similar outcomes were found for genital shape, with natural selection, sexual selection and their interaction all influencing aspects of shape (table 1). For both Relative Warp 2 (RW2) and RW3 there were significant interactions between natural and sexual selection that influenced the evolution of genital shape (figure 4). When considering RW2 and in the absence of natural selection, sexual selection (-N+S) favoured a narrower structure, with the posterior lobe dorso-ventrally elongated and posterior-anteriorly narrowed, but with natural and sexual selection elevated (+N+S) the posterior lobe was dorso-ventrally compressed and posterior-anteriorly thickened, and the tip of the ventral lobe was more upward pointing (figure 4*a*). Under relaxed natural and sexual selection (-N-S) a similarly compressed shape evolved, while with elevated natural selection and relaxed sexual selection (+N-S) a near consensus configuration evolved (i.e. a shape between the two extremes). This indicates that both sexual and natural selection influence aspects of genital shape, but they appear to select genital shape in different directions. Evolution of RW3 was similar in that elevated natural selection in the absence of sexual selection (+N-S) caused genitals to evolve in the direction of the consensus shape and natural and sexual selection again appeared to be selecting genital shape in different directions. Elevated sexual selection with relaxed natural selection (-N+S) favoured a more elongated and narrow-necked posterior lobe and the tip of the ventral lobe was more upward facing, while elevating both natural and sexual selection (+N+S) resulted in a more thickened, dorso-ventrally compressed posterior lobe and the tip of the ventral lobe was more downward pointing (figure 4*b*). Again, elevated natural selection tends to move genitals to a near consensus configuration.

**Table 1:** Results of the MANOVA and post-hoc tests for genital shape.

MANOVA							
*Predictor*				Wilks’ λ		*F*_7,5_	*P*
Sexual selection (S)				0.082		7.97	0.018
Natural selection (N)				0.118		5.32	0.042
Sexual × Natural selection (S × N)				0.047		14.61	0.005
Centroid size (C)				0.168		3.52	0.092
**Univariate ANOVAs**							
	**S**		**N**			**S × N**	
	***F*** **_1,11_**	***P***	***F*** **_1,11_**		***P***	***F*** **_1,11_**	***P***
Warp 1	0.06	0.809	0.36		0.561	4.42	0.064
Warp 2	5.66	**0.036**	1.52		0.244	18.71	**0.001**
Warp 3	1.01	0.337	0.01		0.986	6.57	**0.026**
Warp 4	1.03	0.332	1.36		0.267	0.01	0.981
Warp 5	0.001	0.986	0.001		0.978	1.01	0.337
Warp 6	1.14	0.310	1.55		0.238	0.185	0.675
Warp 7	6.65	**0.026**	5.08		**0.046**	0.21	0.658

Mean Relative Warp Scores for each population (i.e. 4 replicate populations/treatment ) were line means. There were significant effects of sexual selection, natural selection and their interaction on the multivariate combination of Relative Warp Scores 1–7. Univariate post-hoc tests showed which Warps generated the multivariate significance (**bold** ***P*** **-values**). Centroid size was included as a covariate because previous univariate analyses indicated that it was significantly associated with some Relative Warps. However, it was not significant in the MANOVA and hence we do not interpret it further. Removal of centroid size from these analyses increases the strength of selection associations considerably (e.g. the significance level of the sexual selection effect on Warp 7 become *P* = 0.009) and some marginally non-significant results become significant (SxN for Warp 1 and NS for Warp 4). Nevertheless, to be conservative, we retain centroid size in the model.

For RW7 the picture was different in that both sexual and natural selection significantly influenced shape evolution, but their interaction did not. Additionally, in this instance, the influence of natural and sexual selection seemed to be comparable, causing this aspect of shape to evolve in the same direction (figure 4*c*). Here for example, elevated natural selection favoured a sharper medial tip, as did elevated sexual selection, and the +N-S populations essentially converged on the shape favoured in the -N+S populations. Thus when populations evolve with only one form of selection enhanced, the posterior lobe converges on the consensus configuration. Evolving with enhanced natural and sexual selection (+N+S) however, the tip of the posterior lobe becomes longer and sharper, while with relaxed natural and sexual selection (-N-S) the posterior lobe evolves an incurved tip (figure 4*c*).

## Discussion

Our major finding was that genitals evolved through both sexual and natural selection and their interaction. Furthermore, while some aspects of genital form, notably size, seemed to be favoured by both forms of selection, some aspects of genital shape appeared to be favoured by one form of selection and disfavoured by the other. While finding that sexual selection causes genital evolution is largely expected, although rarely shown experimentally, the natural selection claim is likely to cause more debate, especially because we do not fully understand the mechanism(s) involved. Furthermore, it has been suggested that the elevated natural selection treatment (temperature elevation) could have created an environment that has altered sexual selection [20], and hence what we are attributing to elevated natural selection is really due to sexual selection. However, we do not think that increasing temperature, which we used to elevate natural selection, simply alters the form and strength of sexual selection on genitalia for the following reasons. Firstly, males that are more successful in securing mates (more attractive males), are also males that are better sperm competitors [34]. That is, the two bouts of sexual selection favour the same phenotypes. Additionally we have shown with a formal selection analysis of precopulatory sexual selection on male *D. simulans*, that sexual selection does not differ across temperatures (Ingleby et al unpublished) and male attractiveness is consistent across temperatures [35] (albeit across 23–25°C in both cases). So, precopulatory sexual selection is consistent across temperature regimes and the males which do best in the precopulatory arena, do best in the post-copulatory arena too. In sum, this suggests that altering temperature probably does not alter net sexual selection – or at least we have no evidence that it does. Finally, sperm damage through ageing, sperm metabolism and oxidation has been related to various environmental factors including temperature [36], and we know that elevated temperature impairs male fertility in *D. simulans*. Therefore, the genital evolution we document is consistent with natural selection for enhanced male fertility and as discussed above, we know that the temperature elevation increases natural selection on other traits like cuticular hydrocarbons. So despite not knowing the precise proximate mechanisms generating the response to the elevated temperature treatment, on balance we feel our results are consistent with genital evolution by natural selection and sexual selection, and sometimes these two mechanisms of selection reinforce each other and sometimes they oppose each other.

The antagonistic effects of natural and sexual selection on genital morphology have been inferred from patterns of phenotypic variation [14], [16], [19], but this has rarely been demonstrated experimentally (but see [6]). Our data allow us to directly assess the impact of two major sources of selection acting on the genitalia of *D. simulans,* and as predicted [15], we find that some genital shape changes only evolve through sexual selection when natural selection is relaxed. In contrast, elevated natural and sexual selection had a similar effect on the evolution of genital size and one aspect of shape, with larger genital size and the elongated tip of the posterior lobe each favoured by both modes of selection. Consistent with the findings of this study, some previous studies in the seed beetle, *C. maculatus* [6] and the dung beetle, *O. taurus* [4], [5] have also found evidence of genital evolution in response to the opportunity for sexual selection. Furthermore, in the study that most closely resembles our own, the morphology of non-intromittent genital traits was also responsive to the opportunity for sexual selection and natural selection. However, in *C. maculatus*, the greatest difference in male ‘flap length’ and paramere length (their linear morphological measures most likely capturing size variation) occurred in lines with elevated opportunities for sexual selection and contrasting intensities of natural selection [6], whereas, we found that sexual and natural selection both favoured a similar sized genital arch, (also a non-intromittent trait). Perhaps the difference between our results and those of Cayetano et al. (2011) reflects the opportunity for sexual conflict in these systems. In *C. maculatus* there is considerable evidence that sexually antagonistic selection drives genital evolution, whereas, the available evidence in *D. simulans* suggests that sexual interactions are not antagonistic (but see below).

It is possible that the genetic architecture of the genital arch constrains evolutionary responses in such a way that natural and sexual selection appears to be reinforcing. That is, even though the two modes of selection are in opposition to a degree, the major axis of genetic variation constrains responses to selection to one direction. Constraint to genital evolution seems possible as there is little genetic variance for genitalia in several insect models [7], [9], [12], although this is not always the case [4], [37]. We are currently assessing whether the vector of selection and the vector of maximum genetic variation are in alignment in *D. simulans*. Overall, although we find partial support for a theoretical model [15] and verbal arguments that predict that natural and sexual selection may be antagonistic [14], [16], [19], it appears that the net selection driving genital evolution in *D. simulans* is more complex than previously envisaged. Similar results have recently been reported for cuticular hydrocarbons (CHC) too, with sexual and natural selection and their interaction causing CHC evolution [26].

As noted, the proximate mechanisms underlying the naturally selected evolution of genital size and shape of *D. simulans* are unknown. However, during copulation the posterior lobe of the genital arch of *D. simulans* is deeply inserted into the external female genitalia and grasps the oviscape [24]. The ventral lobe also has a grasping role [23], and it has been argued that the larger and broader posterior lobe of *D. simulans* (relative to *D. melanogaster*) confers an advantage in genital coupling [24]. Our finding, that larger genitalia and the pincer-like tips on the posterior lobe evolve under natural and sexual selection is consistent with males using these structures as hold-fasts to grip females and it is possible that an enhanced grasping function is favoured at 27°C due to the increased activity of flies at a higher temperature. Futhermore, the enhanced grasping function of the genitalia may have also been selected if this reduces sperm damage by decreasing the time between sperm production and fertilization [38]. We do not think this is due to altered sexual selection however, for the reasons outlined above, although we cannot definitively exclude this possibility. Rather the manipulation of natural selection in our study involved a very specific, two degree increase in temperature and this pushes flies close to zero fitness because fertility, a trait that is at least partly naturally selected, drops precipitously at temperatures above this. Additionally, the flies had been evolving at a lower temperature for more than 3 years before our experimental populations were established**,** thus we really were exposing flies to strong natural selection, and we saw responses in non-genital traits that were expected under elevated natural selection [26]. Therefore the genital changes may be the result of fertility selection [38]. Another, partial explanation for the increase in genital size at higher temperature may be that the rate of development and growth of genital structures is increased [39]. Temperature sensitivity has also been shown in the aedeagus of *Drosophilia mediopunctata*, although, larger aedeagi were found at cooler temperatures (16.5°C) and smaller aedeagi at warmer temperatures (20°C) [40]. It is not obvious why genital sizes were contrasting in low versus high temperatures. The findings of Andrade et al. (2005) may reflect the different responsiveness of the intromittent organ compared non-intromittent traits. Supportive evidence of this is that we found that the genital arch had a different temperature sensitivity compared to other morphological traits that we measured as fly size more generally did not differ between temperature exposures. An additional possibility is that natural selection on other traits that are genetically correlated with genital size resulted in the naturally selected changes in genital morphology [41]. This would be particularly true if the nature of pleiotropy changed with temperature [38]. However, the evolution of genital size and one aspect of shape were similarly influenced by elevated sexual selection, so these configuration changes apparently confer naturally and sexually selected advantages, and the available evidence is that genitals are often genetically uncoupled from other characters [7], [9], [12].

Our experimental evidence that male genitalia evolve through sexual selection is clear. Models of sexual selection suggest that genitalia may evolve through antagonistic interactions between males and females, cryptic female choice for copulatory courtship or sperm competition [2]. Distinguishing between these different mechanisms of sexual selection is challenging, particularly in the light of the apparent interplay between sexual and natural selection we find here. Nevertheless, it is generally agreed that cryptic female choice and/or sperm competition are primarily responsible for genital evolution [1], [2], [42], [43], and sperm competitiveness is heritable [27] and likely to be influenced by genital form in *D. simulans*. Additionally, recent investigations in *Drosophila* raise the possibility that male genital wounding may enhance male post-copulatory fitness [44]. During copulation, parts of the aedeagus are opened, penetrating and wounding the walls of the female genital tract [44]. Interestingly, the sites of wounding correspond to the areas were the posterior lobe grips the female abdomen externally [44]. It is not clear precisely how wounding elevates male fitness, but there is some evidence that harmful males are superior sperm competitors in *D. melanogaster* [45]. Investigations to determine precisely how genital variation influences male sexually selected fitness in *D. simulans* are ongoing, but to date we have no evidence that high mating frequency harms females [30].

In conclusion, we provide compelling experimental evidence that genitals evolve through the combined effects of natural and sexual selection. We show that some aspects of the genital shape were favoured by sexual and opposed by natural selection, whereas for others, natural and sexual selection had seemingly complementary influences. Given the near ubiquity of complex genitalia, it is perhaps unsurprising that the underlying selection influencing genital form is complex in its action and does not always involve antagonistic natural and sexual selection.
